# Interacting effects of land use and climate on rodent-borne pathogens in central Kenya

**DOI:** 10.1098/rstb.2016.0116

**Published:** 2017-04-24

**Authors:** Hillary S. Young, Douglas J. McCauley, Rodolfo Dirzo, Charles L. Nunn, Michael G. Campana, Bernard Agwanda, Erik R. Otarola-Castillo, Eric R. Castillo, Robert M. Pringle, Kari E. Veblen, Daniel J. Salkeld, Kristin Stewardson, Robert Fleischer, Eric F. Lambin, Todd M. Palmer, Kristofer M. Helgen

**Affiliations:** 1 Department of Ecology, Evolution and Marine Biology, University of California, Santa Barbara, CA 93106, USA; 2 Mpala Research Centre, Box 555, Nanyuki, Kenya; 3 Department of Biology, Stanford University, Stanford, CA 94305, USA; 4 Department of Evolutionary Anthropology, Duke University, Durham, NC 27708, USA; 5 Duke Global Health Institute, Duke University, Durham, NC 27710, USA; 6 Center for Conservation Genomics, Smithsonian Conservation Biology Institute, National Zoological Park, Washington, DC 20008, USA; 7 Mammal Section, National Museums of Kenya, Nairobi, Kenya; 8 Department of Anthropology, Purdue University, West Lafayette, IN 47907, USA; 9 Department of Human Evolutionary Biology, Harvard University, Cambridge, MA 02138, USA; 10 Department of Ecology and Evolutionary Biology, Princeton University, Princeton, NJ 08544, USA; 11 Department of Wildland Resources and Ecology Center, Utah State University, Logan, UT 84322, USA; 12 Department of Biology, Colorado State University, Fort Collins, CO 80523, USA; 13 Department of Earth System Science and Woods Institute for the Environment, Stanford University, Stanford, CA 94305, USA; 14 Department of Biology, University of Florida, Gainesville, FL 32611, USA; 15 Division of Mammals, National Museum of Natural History, Smithsonian Institution, Washington, DC, USA; 16 School of Biological Sciences, University of Adelaide, Adelaide, South Australia 5005 Australia

**Keywords:** disease, diversity, dilution effect, susceptible host regulation, landscape ecology, land-use change

## Abstract

Understanding the effects of anthropogenic disturbance on zoonotic disease risk is both a critical conservation objective and a public health priority. Here, we evaluate the effects of multiple forms of anthropogenic disturbance across a precipitation gradient on the abundance of pathogen-infected small mammal hosts in a multi-host, multi-pathogen system in central Kenya. Our results suggest that conversion to cropland and wildlife loss alone drive systematic increases in rodent-borne pathogen prevalence, but that pastoral conversion has no such systematic effects. The effects are most likely explained both by changes in total small mammal abundance, and by changes in relative abundance of a few high-competence species, although changes in vector assemblages may also be involved. Several pathogens responded to interactions between disturbance type and climatic conditions, suggesting the potential for synergistic effects of anthropogenic disturbance and climate change on the distribution of disease risk. Overall, these results indicate that conservation can be an effective tool for reducing abundance of rodent-borne pathogens in some contexts (e.g. wildlife loss alone); however, given the strong variation in effects across disturbance types, pathogen taxa and environmental conditions, the use of conservation as public health interventions will need to be carefully tailored to specific pathogens and human contexts.

This article is part of the themed issue ‘Conservation, biodiversity and infectious disease: scientific evidence and policy implications’.

## 1. Introduction

Given the burgeoning global burden of zoonotic disease [1–4] and a hypothesized protective effect of biodiversity on disease prevalence and exposure risk [5–7], there is growing interest in the possibility that biodiversity conservation may reduce the impacts of infectious disease on human populations [8]. However, the spread of infectious agents across species—including to humans—arises from complex biotic and abiotic interactions [9]. Thus, the impacts of anthropogenic perturbations (and their mitigation via conservation interventions) on pathogen assemblages and disease risk are unlikely to be a simple function of community diversity; instead, they likely reflect multi-factorial interactions that are difficult to predict [10,11].

Research on the effects of disturbance has typically focused on single pathogen taxa and particular types of anthropogenic disturbance, and overall results have been mixed. For instance, deforestation has been shown to both increase and decrease risk of malaria [12,13]. Likewise, the effects of forest loss and fragmentation on Lyme disease prevalence seem to be strong at some scales and contexts, but absent or reversed in others [14]. Invasive plant species have been shown to increase risk of Lyme disease in some areas [15], but have also been shown to decrease survivorship of disease-transmitting ticks in other regions [16]. Similarly, while size-selective loss of large wildlife has been shown to increase landscape risk for flea-vectored, rodent-borne pathogens [17], it has also been shown to decrease the abundance of questing ticks critical to the transmission of tick-borne pathogens [18].

In an effort to identify potentially general relationships between anthropogenic perturbations and pathogen abundance, we investigated the effects of three pervasive forms of anthropogenic disturbance on landscape-level abundance of rodent-borne pathogens in semi-arid East Africa savannahs. Specifically, we examined the effects of large-wildlife declines [19] and the conversion of savannah habitat to both cropland and intensively used livestock pasture [20] on the ecology of five locally important rodent-borne pathogen genera; *Anaplasma, Bartonella*, *Theileria*, *Borrelia* and *Hepatozoon*. Many taxa of these pathogens are regionally important to livestock, wildlife and human health [21–24]. While the detailed ecology and life histories of many of the specific pathogens studied here are poorly known, these genera represent a diversity of transmission modes: (i) primarily flea-borne transmission (*Bartonella*), (ii) primarily hard tick-borne transmission (e.g. *Theileria, Anaplasma*), and (iii) primarily lice and soft-tick transmission (local species of *Borrelia*). Additionally, while most are transmitted via the bite of the vector, *Hepatozoon* is likely transmitted via ingestion of the vector. Some of these species and strains likely use rodents as primary reservoirs (e.g. *Bartonella*), whereas others may use rodents only incidentally (e.g. many strains of *Theileria*) with large mammals (including domestic animals) as primary hosts. Given the variation among these pathogens in reservoir hosts, vectors and transmission strategies (see electronic supplementary material, S1), this study provided the opportunity to test whether a systematic effect of disturbance on pathogen abundance exists across savannah landscapes for one broad group of pathogens (rodent-borne pathogens), or whether this relationship was instead idiosyncratic across pathogens or disturbance types.

We focus on rodent-borne pathogens, because rodents are among the most diverse and numerically abundant vertebrate taxa in the world [25]. Rodents are also the most important taxa as reservoirs of important zoonotic disease in the region [26–28], with a recent review identifying at least 217 rodent species as reservoirs for 66 known zoonoses globally, including 79 species that are established as hyper-reservoirs of 2–11 zoonotic infectious agents [27]. The burden of rodent-borne disease in East Africa is thought to be particularly high, including cases of brucellosis, leptospirosis, plague, rat-bite fever, murine typhus, tick typhus, tularaemia and relapsing fever [28,29]. As a group, rodents and other small-bodied mammals are thought to be relatively robust to anthropogenic disturbance, with many species actually increasing in abundance following anthropogenic disturbance in land use [30], potentially driving systematic patterns of increase in this group of infectious diseases.

For rodent-borne pathogens, changes in host species composition and abundance following seasonal or interannual variation in climate have been shown to alter prevalence or risk of hantavirus pulmonary syndrome [31], leptospirosis [32] and plague [33,34]. Indeed, climate change is broadly considered a major likely driver of changes in zoonotic disease patterns [35], both directly and indirectly, including through changes in water availability [36]. The effects of climate change may be particularly strong for vector-borne pathogens, such as those in this study, because the vectors themselves are often highly sensitive to changes in temperature or precipitation [37,38].

We therefore conducted this work along a precipitation and productivity gradient in central Kenya to explore both the independent and interacting effects of climate and land-use change on density of animals infected with these pathogens. Moreover, theoretical and empirical data suggest that these drivers are likely to interact [9]. Climate and productivity are known to modulate many effects of disturbance on the abundance, composition and interactions between species [39–41]. Indeed, previous work in our Kenyan study system has shown that effect size of land-use change and wildlife loss on both plant and rodent communities hinges on local environmental context [42–44]. In particular, the effects of cropland and pastoral land-use conversion on small mammal abundance seem to vary in both magnitude and direction based on climatic context [39]. Here we build on this work to test the importance of the climate gradient in driving changes in abundance of infected animals, and to assess whether local climate conditions interact with disturbance to change the magnitude or even direction of the effect of disturbance on pathogen prevalence.

## 2. Methods

### (a) Study site

Research was conducted in Laikipia County, central Kenya, because East Africa is considered to be a hotspot for emergence and re-emergence of zoonotic disease [1], and is also a hotspot of mammalian diversity [45]. Thus, any effects of disturbance on disease mediated through mammal host diversity would be likely to emerge in this system. In addition, East Africa in general, and central Kenya in particular, is experiencing multiple simultaneous forms of rapid land-use and climatic change. A fast-growing and increasingly settled population has driven high recent rates of land-use conversion, sharp increases in abundance of domestic livestock [46–49], and rapid declines in abundance and diversity of large wildlife in many regions [50–52]. While climate models vary in their predictions of future climate regimes, particularly for precipitation, ‘long rains’ have already decreased 20–30% from the 1950 to 1979 totals, driving a generally warmer and drier climate in the region; in addition, locally specific precipitation models predict strong changes in precipitation patterns to continue [53–56].

Fieldwork was conducted between January and July 2011, across 92 sites spanning a 3000 km^2^ area of semi-arid savannah ecosystem throughout Laikipia. To maximize control of spatial and temporal variation, these sites were sampled using a paired design, with each pair consisting of one conserved and one disturbed site located in close (less than 1 km) physical proximity and sampled simultaneously, and at least 2 km from the nearest neighbouring site of a pair. Conserved sites consisted of large privately owned ranches, which had wildlife conservation as a primary management goal. Disturbed sites included three types of human activity: (i) community-owned ranches with extensive pastoral use (goats, cattle or camels), (ii) small- and large-scale cropland (agricultural) and (iii) isolated large-wildlife removal (defaunation) achieved via electrified fences (the Kenya long-term exclosure experiment (KLEE) and the ungulate herbivory under rainfall uncertainty (UHURU) experiment). Both KLEE and UHURU provide controlled, replicated exclosure defaunation experiments [57,58]. *A priori* assessments of disturbance type were subsequently confirmed via (i) field measurements of wildlife and domestic stock activity [43,44] and (ii) plant community composition on each site [43,57]. Each of the sites surveyed was 1 ha in size (100 × 100 m).

The nested landscape design of this experiment should be appropriate to the scales at which both biological interactions and environmental variation are thought to shape disease dynamics [59]. Our study sites were spread across strong environmental gradients in both mean annual precipitation and soil type. Mean annual precipitation ranged from approximately 300 to approximately 900 mm rainfall per year, interpolated from a series of 75 long-term rainfall gauges in the region [60]. Quantity and timing of rainfall events are the main drivers of primary productivity in grassland ecosystems, and in this region have been shown to be tightly correlated with both understory biomass and NDVI for a series of sites [57].

### (b) Field sampling

At most study sites, we surveyed small mammal communities using a 100 × 100 m grid of Sherman traps composed of 100 traps, each of them 10 m apart. However, at 18 sites (the UHURU experimental large-wildlife removal plots), we used only 49 traps in the interior 0.5 ha of the 1 ha site (again on a 10×10 m grid) to minimize edge effects. Relative small mammal abundance (including rodents, shrews and elephant shrews) was estimated, using catch per unit effort (details in Young *et al*. [44]). Animals were identified to species using a combination of morphological and genetic approaches. Ectoparasites were combed off animals using a standardized number of sweeps on one side of each animal, counted and identified to species [44].

### (c) Pathogen sampling

DNA from 884 individual small mammals (about half of all individuals captured), representing 23 species was extracted from blood samples (either frozen or dried on Whatman FTA^®^ cards; GE Healthcare Lifesciences, Piscataway, NJ) using QIAGEN (Valencia, CA) blood and tissue kits according to manufacturer's protocols. To ensure data reliability, replicate samples of DNA were extracted from a subset of individuals either two (*n* = 174) or three times (*n* = 5). We screened 40–80 individuals per trapped species whenever possible, with the exception of species where fewer than 40 total individuals were captured over the course of the study (e.g. *Dendromus mysticalis, Dasymys incomtus, Rattus rattus, Lemniscomys striatus, Zelotomys hildegardeae, Mus sorella, Arvicanthis niloticus, A. nairobae, Paraxerus ochraceus, Xerus erythropus*)*,* for which we screened all individuals. Individuals used for screening from commonly captured species were randomly selected within a land-use type, with the number of individuals sampled per land-use type proportional to the frequency of occurrence of that species in that land use. For *Crocidura* spp. (shrews, 3% of total captures), all species were considered together for screening because of difficulties in accurately identifying species in the field, problems with preserving DNA from these species for genetic analysis and low sample sizes.

Based on known patterns of pathogen occurrence in the region [61], we screened small mammals for the following pathogens: *Anaplasma*, *Babesia*, *Hepatozoon*, *Theileria*, *Bartonella*, *Borrelia*, *Coxiella burnetii* and *Rickettsia*. We screened for all pathogens using polymerase chain reactions (PCR) following Campana *et al*. [61]. All positive apicomplexan and *Coxiella* PCR products, and a representative sampling of positive *Anaplasma*, *Bartonella Borrelia* and *Rickettsia* products were Sanger sequenced on an ABI 3130 (Life Technologies, Carlsbad, CA). Sequences were edited manually using Sequencher5 (Gene Codes Corporation, Ann Arbor, MI). Contaminants were identified by alignment to the GenBank non-redundant nucleotide database using Megablast [62]. Edited sequences were aligned to publicly available reference sequences (downloaded from GenBank during September–October 2014) in Geneious 8 (Biomatters Ltd., Auckland, New Zealand). Strains were classified based on maximum-likelihood trees constructed in Geneious using the GARLI [63] plugin under default settings and the RAxML plugin [64] under the GTR + γ + I model, with 100 bootstrap replicates. Some strains could not be classified owing to either mixed infections (represented by the presence of multiple peaks in the sequence traces at diagnostic sites) or low-quality traces.

### (d) Statistical analyses

We first tested for variation in prevalence (proportion of individuals infected) within species by disturbance status. We performed this analysis for the 12 species where both (i) more than 40 individuals were screened and (ii) more than 10 of the tested individuals occurred in each of at least two habitat types (conserved, cropland, pastoral and wildlife removed). To do this, we used a generalized linear mixed-effects model (GLMMER) for each species with infection status as the response variable, and land-use type as the explanatory factor, using a negative-binomial distribution with the lme4 package in R [65]. This analysis was repeated for each species–pathogen combination.

Next, in order to specifically identify drivers of variation in landscape-level pathogen abundance (estimated as the total number of infected animals per hectare), we constructed GLMMERs for each pathogen and for total abundance of fleas (ticks and lice were relatively uncommon, with less than 5% prevalence across individuals, and were potentially not well sampled with our methods). Our response variable was the estimated number of infected animals per hectare (or the total number of fleas per hectare) and the following variables were used as explanatory factors: land-use type (conserved, wildlife removed, pastoral and cropland), rainfall (mean annual values) and the interaction of rainfall and land-use type (based on results from prior studies [38,39]). Given the high frequency of zeros in our dataset, we used the Tweedie distribution family to model the distribution of the data. Tweedie has been shown to be effective at numerically computing likelihoods (as a compound Poisson-gamma distribution [66,67]) when data are a mixture of skewed continuous values and many zeroes (e.g. as in the case of seasonal rainfall [68]). To account for correlations in the matched pairs design of sampled plots, we used a random paired ID grouping variable. In addition, we used correlograms and the Moran's I statistic to estimate the autocorrelation among sites as a function of increasing distance [43]. We tested for spatial independence by computing Moran's I at sequential distance lags greater than distances between paired plots. Because results revealed positive and significant spatial autocorrelation for several of our responses, the analysis was implemented as a GLMMER using the glmmPQL function [69]. We used this approach because it can simultaneously model Tweedie distributed data, account for the matched pair design as a grouping variable, and account for spatial autocorrelation through generalized least-squares (GLS; [70]). We used the exponential variogram procedure, to model the spatial dependence in the data. We subsequently performed a second GLMMER, again using spatially corrected data and site pair as a grouping variable, to explore the mechanisms behind this relationship. For this analysis, we used site-level small mammal diversity (measured using Shannon's diversity index) and site-level mammal density (catch per trap night) as explanatory variables, and number of individual small mammals infected with each pathogen as response variables.

To explore the hypothesis that systematic changes in rodent community composition might drive changes in landscape abundance of pathogens following disturbance, we first tested for a nested relationship within host species using weighted nestedness, a metric developed by Galeano *et al.* [71] that takes into account the abundance of animals instead of presence–absence matrices. After documenting significant levels of nestedness, we ranked the species in order of their generality across sites. As a field-based metric of robustness to disturbance, we calculated the proportion of individuals captured in disturbed habitats for each species. Given that all trapping efforts were paired in both space and time across disturbed and conserved habitats, this provided a metric of the ability of each species to persist in disturbed habitats. We then used linear models to quantify how prevalence of infection (arcsin-transformed) per species varied as functions of (i) species robustness to disturbance (proportion of all individuals per species that were captured in disturbed habitats) and (ii) species relative abundance (as proportions of total individual animals captured). Given the low prevalence of some of the pathogens screened, these analyses were performed excluding the most infrequently sampled species (i.e. those with fewer than 20 individuals captured and screened), based on the conservative assumption that estimates of pathogen prevalence were not reliable with such small sample sizes. We chose 20 individuals for this break because it represented a natural break in the data across species. To account for non-independence among species owing to variation in the effect of shared ancestry among hosts, we used phylogenetic generalized least-squares models [72] in the R package CAPER [73]. The tree used was generated using mitochondrial CO1 from individuals collected in this study (details in Dormann *et al*. [74]). CO1 has been established as a broadly reliable indicator of phylogeny in other African rodents [75].

## 3. Results

### (a) Small mammal communities

**Figure 1.**
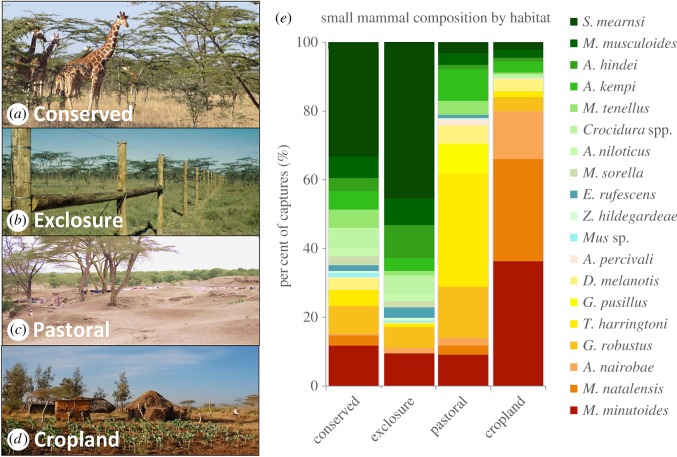
Four land-use types across Laikipia, Kenya were included in this analysis: (*a*) conserved sites, (*b*) experimental exclosure sites (where large mammals were excluded with electrified fences), (*c*) pastoral land use with heavy grazing of domestic stock and (*d*) cropland sites with small- or large-scale agricultural crops. Community composition of small mammals varied strongly across land-use types (*e*). For visual clarity, rare species (less than five individual captures) are not depicted in panel (*e*).

**Figure 2.**
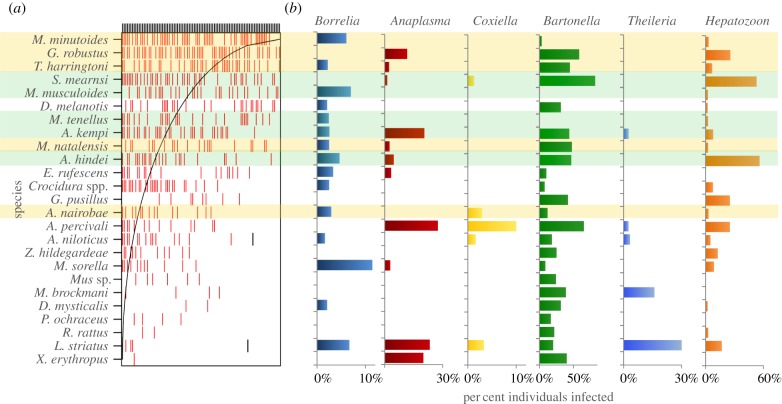
Panel (*a*) shows the presence per site of each small mammal species. Rows are species and columns are sites; the presence of each species at a given site is depicted with a red vertical bar. (Nestedness plot constructed using the ‘nestedtemp’ function in the ‘vegan’ package.) This reveals a strongly nested assemblage structure, with some species (e.g. *M. minutoides* and *G. robustus*) present at both higher diversity sites (on left) and lower-diversity sites (on right), and other species present only in a subset of relatively high-diversity sites (e.g. *L. striatus*). The grey ‘boundary line’ shows expected distribution if species were perfectly nested, such that absences above and to the left of the line, and presences below and to the right of the line are both unexpected with a perfectly nested distribution. If species that were highly generalist across sites also tended to have more pathogens, we would expect that the nested structure would correlate with the proportion of infected individuals for each of the pathogens detected (high prevalence expected at top of the graph); however, we see no evidence of this (*b*). The five species most characteristic of cropland and pastoral habitats are highlighted in gold, whereas the five species most characteristic of conserved and exclosure habitats are highlighted in green.

We captured 1710 individuals representing 29 small mammal species (*Acomys kempi, A. percivali, Aethomys hindei, Arvicanthis nairobae, A. niloticus, Crocidura* spp., *Dendromus melanotis, D. mysticalis, Dasymys incomtus, Elephantulus rufescens, Gerbillus pusilus, Gerbilliscus robustus, L. striatus, Mastomys natalensis, Mus minutoides, M. musculoides, M. sorella, M. tenellus, Mus* sp., *P. ochraceus, R. rattus, Saccostomus mearnsi, X. erythropus* and *Z. hildegardeae*). Six of these species were *Crocidura* spp. shrews (final identifications outstanding), which are hereafter pooled in all analyses. Most species showed some degree of habitat specialization [44], suggesting that the small mammal communities were highly differentiated among habitats (figure 1). Although habitat specialists were found in every land-use type, we observed a nested overall species-assemblage pattern (matrix temperature = 26.8, *p* < 0.01; figure 2*a*), such that sites with fewer rodent species typically contained a subset of the species present in more diverse sites (figure 2*a*).

**Figure 3.**
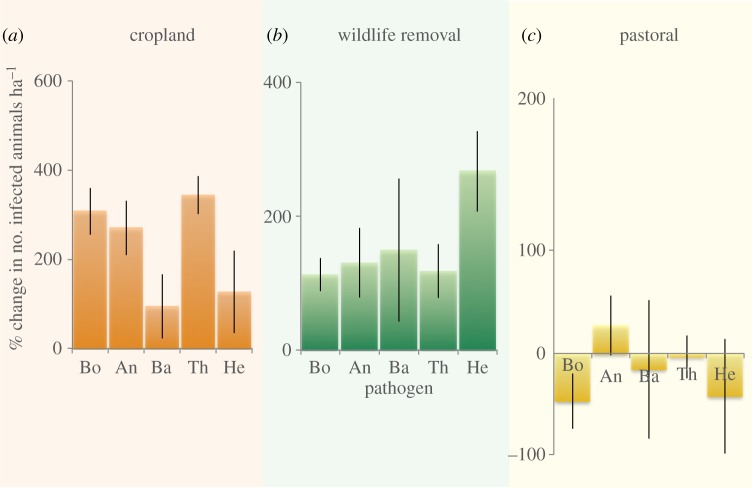
The net effect of three types of disturbance (relative to conserved landscapes) on number of infected small mammals per hectare for each of the five most commonly detected pathogens across all sites (±s.e.). Pathogens are abbreviated as follows: Bo, *Borrelia*; An, *Anaplasma;* Ba, *Bartonella*; Th, *Theileria* and He, *Hepatozoon.* Note differences in scale across panels.

While data on richness, diversity and abundance of small mammals by habitat type and climate are fully considered in Young *et al*. [44], summary statistics are provided in electronic supplementary material, table S1. To review these previous findings here, overall small mammal abundance increased significantly (roughly doubled) in exclosure (large-wildlife removal) treatments. However, there were no significant changes in average abundance in cropland and pastoral land use when compared with conserved land use, although there were significant (positive and negative) changes in abundance in some climatic conditions. These patterns were inverted for metrics of diversity and richness. Specifically, exclosure treatments did not significantly alter small-mammal community structure or diversity metrics, but both pastoral and cropland land-use conversion were correlated with significant reductions in the richness of small mammals (estimates of 5.22 and 7.83 in conserved and exclosure habitats, respectively, versus 4.24 and 4.38 in pastoral and cropland habitats, respectively), as well as strong changes in species composition. Both within and across land-use types, precipitation was positively correlated with small mammal density [44].

### (b) Pathogen prevalence across and within small mammal species

Among 884 individuals that were screened, we found animals infected with *Bartonella* (*n* = 286), *Hepatozoon* (*n* = 116), *Anaplasma* (*n* = 38), *Borrelia* (*n* = 24), *Theileria* (*n* = 22) and *Coxiella burnetii* (*n* = 8) (electronic supplementary material, S1). No positive samples of *Rickettsia* or *Babesia* were found in small mammals despite positive screenings of these taxa from questing ticks hosted by these small mammals in this region using the same primers (H.S.Y. 2016, unpublished data). For *Bartonella*, we identified at least 17 major strains (some with substrains) in the 226 sequenced PCR products (electronic supplementary material, S1 and figure S1). Approximately 10% of samples sequenced had multiple strains detected. For *Anaplasma,* we found two strains that were separated by a single nucleotide polymorphism. Unfortunately, this marker does not permit species-level identification, matching several species with more than 99% nucleotide identity. We found one *Theileria* strain most closely related to *T. equi* (however, this had only 89% similarity)*.* We found two major *Hepatozoon* strains (both with substrains), one most closely related to *H. arygbor* and *H. erhardovae* and the other most closely related to *H. felis* and *H. ursi* (electronic supplementary material, figure S2). All *Borrelia* sequences had 100% nucleotide identity to *Borrelia burgdorferi*. However, flagellin is a conserved genetic region, and it is likely this is another *Borrelia* species. Owing to the very low prevalence of *Coxiella burnetii* in the samples, it was excluded from all subsequent analysis*.* For all other analyses, we pooled strains within genera. For none of the 12 small-mammal species with robust sampling across multiple habitat types did we detect significant differences in prevalence across land-use types for any pathogen taxon *within* a host species (using a Bonferroni-corrected alpha of *p* = 0.01).

Although striking variation in prevalence was observed for all pathogens across species, only for *Theileria* was there a significant relationship between robustness to disturbance (proportion of total captures in disturbed habitats per species) and species-level prevalence for the pathogen *across species* in our phylogenetic comparative analyses (*F* = 5.45, *p* = 0.04, *R*^2^ = 0.22). In this case, species that were abundant in disturbed habitats appeared to have higher average prevalence of *Theileria.* However, this effect was strongly driven by a single species—the high prevalence of *Theileria* in *Lemniscomys striatus,* a cropland habitat specialist—and when this species was removed the correlation between robustness to disturbance and species-level prevalence was no longer significant. We also did not detect a significant relationship between a species' overall relative abundance (proportion of total number of individuals captured over the entire course of the study) and prevalence for any pathogen.

### (c) Land-use effects on abundance of infected rodents

For all five pathogens detected in more than eight individuals, the number of infected rodents increased significantly (between 1.7- and 5.8-fold increase) in cropland sites relative to paired conserved sites (figure 3 and electronic supplementary material, table S2). Similarly, for four of these five pathogen taxa (all except *Anaplasma*), the total number of infected rodents was significantly elevated in experimental sites with large wildlife excluded compared with paired control sites where large wildlife were present. Results were more mixed in pastoral sites. For three of the five pathogen taxa, we found no significant difference in the number of infected rodents between pastoral and paired conserved sites (*Bartonella*, *Theileria* and *Hepatozoon*). One of the pathogens (*Anaplasma*) showed a statistically significant, albeit relatively modest (27%), increase in pastoral relative to conserved sites (figure 3 and electronic supplementary material, table S2), whereas the fifth taxon, *Borrelia*, significantly decreased in pastoral relative to conserved sites.

Site-level Shannon diversity index of the small mammal community was positively correlated with absolute number of infected animals for two pathogens: *Bartonella* (*Z* = 0.27, *p* = 0.04) and *Hepatozoon* (*Z* = 0.64, *p* < 0.0001), and there was no significant relationship with number of infected animals for the other two pathogens (electronic supplementary material, figure S3 and table S3). Shannon diversity of small mammals was not correlated with abundance of small mammals per site within any habitat type (electronic supplementary material, figure S4). Small mammal density was, unsurprisingly, significantly correlated with density of infected animals for all pathogens; these effects tended to be stronger in cropland sites (electronic supplementary material, figure S5 and table S3).

### (d) Climate and climate–land use interaction effects at landscape scale

**Figure 4.**
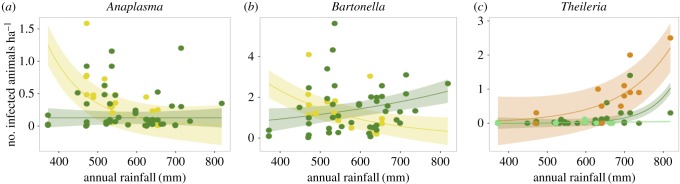
For three of the five pathogens examined (*Anaplasma* (*a*), *Bartonella* (*b*) and *Theileria* (*c*)), there were significant interactions between the effect of land-use type and that of mean annual rainfall on number of infected small mammals per hectare. For *Anaplasma* and *Bartonella*, the number of infected animals decreased with rainfall in pastoral (yellow) sites but were uncorrelated or positively correlated with rainfall in conserved (dark green) sites. For *Theileria,* the number of infected animals increased with rainfall overall, but this effect was stronger in cropland (orange) than conserved sites and was not detected in wildlife exclosure (light green) sites. Shading represents 95% confidence intervals.

There was only a significant main effect of rainfall on the number of *Theileria-*infected rodents (electronic supplementary material, table S2). This relationship was positive, with more infected small mammals present in high rainfall when compared with low rainfall sites (*p* < 0.01). For *Anaplasma, Theileria* and *Hepatozoon*, we found significant interactions between land-use type and rainfall on the number of infected small mammals (figure 4). The direction and locations of this interaction varied among pathogens: for *Anaplasma* and *Bartonella*, the number of infected individuals decreased with rainfall in pastoral sites but were uncorrelated (*Anaplasma*) or increased (*Bartonella*) with rainfall in conserved sites. In contrast, for *Theileria,* we observed a strong and nonlinear increase with rainfall in cropland sites, which was weaker in conserved sites and absent in exclosure sites. For flea abundance (total number of fleas per site), results closely paralleled those seen for *Bartonella,* the pathogen most likely to be transmitted by fleas in this landscape (electronic supplementary material, figure S6), with the number of fleas decreasing with rainfall in pastoral sites but increasing with rainfall in conserved sites (electronic supplementary material, table S4).

## 4. Discussion

Our results suggest that some forms of anthropogenic disturbance can cause systematic increases in landscape-level abundance (density of infected animals) of rodent-borne pathogens; however, the magnitude of the impact depends on the pathogen and the type of disturbance. Specifically, conversion to cropland and experimental removal of large wildlife tended to cause strong increases in density of infected small mammals. In comparison, pastoral land use, one of the most regionally and globally widespread types of land-use change [20,76], exerted little if any effect on density of infected animals. Given that prior studies have shown that land use has little effect on the density of fleas per animal in this system [74,77], this likely correlates fairly directly to landscape-level risk, at least for flea-borne pathogens.

Annual precipitation across these sites has been shown to have strong effects on both plant and small mammal community composition, and increases the total abundance of both plants and, indirectly, small mammals [43,44,57], presumably via bottom-up stimulus to the system. Yet this climatic variable had surprisingly little direct effect on the number of infected rodents per site. For only one pathogen, *Theileria,* was there any main effect of precipitation on number of infected rodents per hectare. This finding is consistent with prior analyses of ectoparasites in this region, which failed to find a strong effect of precipitation on prevalence and intensity of small mammal fleas that transmit many of the pathogen taxa examined in this study [74], and consistent with results of other regional studies on these tick-borne diseases in East Africa [78].

However, while the main effect of precipitation was not pronounced, we found significant interactions between rainfall and disturbance for three pathogen taxa that exhibited significant correlations with rainfall in at least one type of disturbed landscape. For instance, while pastoral land-use change did not significantly affect the number of infected animals for either *Anaplasma* or *Bartonella*, for both pathogens we found that pastoral land use modified the impact of precipitation. In low-precipitation areas, the number of infected animals per hectare was greater in pastoral than conserved sites, whereas for *Bartonella*, in high-rainfall sites the reverse was observed. For *Theileria*, we observed a particularly pronounced increase in the number of infected animals with rainfall in cropland sites. These results are similar to results from mammal and plant communities in the region, which showed that both the magnitude and direction of the effect of various types of anthropogenic disturbance varied with environmental context [43,44,57]. Regionally, it suggests the potential for interactions between land-use change and climate change on zoonotic disease risk.

The mechanisms underlying these context-dependent relationships between land-use change and density of infected rodents are not immediately obvious, and are likely varied, owing to a combination of changes in density and competence across sites (electronic supplementary material, figure S5). While recent research has suggested that commensal, typically fast-lived species are likely to drive higher prevalence in disturbed contexts [27,79–81], we failed to observe any systematic evidence of this phenomenon. However, compositional change of particular species did appear to be important, at least in cropland systems. In particular, we found that the commensal and fast-living species *Mastomys natalensis,* which is well known as a hyper-reservoir of zoonotic disease, has both relatively high prevalence of infection (figure 2) and is nearly exclusively found in cropland systems [82]. As in other systems [83], this species may drive much of the effect of cropland disturbance on abundance of infected individuals. Cropland conversion does not cause systematic increases in small mammal density across all sites [44], so compositional changes rather than density likely explain these results.

In contrast to the potential importance of such compositional changes in cropland sites, in sites where large wildlife has been removed, small mammal composition was largely unchanged [17,44,57]. In these sites, the effect of land use is instead likely owing to well-documented increases in total small mammal abundance following large-wildlife removal or loss, as small mammals are released from competition for food with their larger counterparts [57,84–86]. Notably, across sites, the effect of disturbance on number of infected animals was not negatively correlated with host richness or diversity *per se* for any of the pathogens examined, and indeed for three pathogens showed a weak positive correlation.

In general, pastoral land use also does not seem to cause such strong and systematic changes in plant or small-mammal abundance as does experimental wildlife removal [17,43], likely because (at least at low-to-moderate densities) domestic herbivores often closely mimic and at least partially compensate for many effects of native large herbivores [87,88]. In this case, domestic herbivores may substitute for native large herbivores as food sources for vectors. The compositional changes of small mammals in response to pastoral use are also less dramatic [44,87]. Pastoral landscapes also do not tend to have the highly seasonal compositional dynamics that cropland does in the region; thus, the compositional changes in small mammals that do occur in pastoral systems may not favour the very fast-living, commensal rodents that are most likely to be hyper-reservoirs of infection [27].

Notably, while our results are generally consistent with the prevailing hypothesis that anthropogenic disturbances (at least cropland conversion and large-wildlife declines) tend to lead to increases the abundance of infected animals [89], our results do not seem clearly consistent with the dilution-effect hypothesis. As originally defined [6], this hypothesis argues that as local biodiversity decreases, pathogen prevalence (and abundance) should increase, owing to systematic loss of less competent hosts in low diversity landscapes. However, in this study, we observed (i) either no correlation or weak positive correlations (an ‘amplification effect’) between local-scale small mammal diversity and abundance of infected mammals, and (ii) no systematic relationship between proportion of infected individuals within a host species (a proxy for species competence), on the one hand, and the likelihood of that species persisting in disturbed landscapes, on the other hand. Yet, because many of these pathogens have hosts beyond small mammals, it is possible that such a relationship might emerge if other metrics of diversity (e.g. all mammals, irrespective of size) were used. Notably, in sites where wildlife has been removed, we do see evidence for susceptible host regulation [6,14], with the systematic release of small mammal hosts following removal of large mammals.

In addition to the effects of land-use conversion on many of these vector-borne pathogens via changes in host dynamics, many of the changes in abundance of infected hosts may also be due to factors that are unrelated to composition or density of small mammal host assemblages (e.g. direct effects of climate on vectors). Certainly, climate is well established to directly impact survivorship and growth rates of both ticks and fleas [90–94], and microclimates will be strongly impacted by land-use change (e.g. less ground cover in pastoral sites may create more desiccating habitats). Changes in the abundance and composition of other hosts (large- and medium-sized mammals, reptiles and birds) also are known to occur when land use changes in this system [16,79], and may also strongly impact survivorship and reproductive rates for many vectors. Thus land-use change may affect growth, survivorship and reproduction of these vectors independently of any effects on small mammals.

Collectively, our results suggest that, regardless of mechanism, we can expect increases in abundance of rodent-borne pathogens at landscape scales following both conversion to cropland and the elimination of large wildlife. The effects of pastoral conversion and wildlife displacement are weaker. For several pathogens, we found strong interactions between disturbance and climate, consistent with synergies between these forms of disturbance. These effects do not appear to be driven by changes in diversity or richness of small mammal communities following disturbance, but rather by shifts in abundance and compositional changes following conversion to cropland and large wildlife loss. However, there may well be other mechanisms that also drive these changes. On the one hand, this suggests that conservation can be an effective tool for pathogen reduction in some contexts (e.g. wildlife loss *per se*, as has occurred in some parts of Africa following human conflict and/or intensive hunting [90]). However, the observed contingencies between different forms of disturbance and precipitation in this semi-arid region suggest that the compounded effects of multiple anthropogenic global changes may be difficult to predict. Further research aimed at identifying the mechanistic bases of these interactive effects will be essential to produce accurate forecasts of zoonotic disease risk in rural areas subjected to both local transformations of land use and a globally changing climate.

## Supplementary Material

Supplementary material: Pathogen identification and ecology
