# Supplementary material for: Interacting effects of land use and climate on rodent-borne pathogens in central Kenya
Source: Philos Trans R Soc Lond B Biol Sci. 2017 Apr 24;372(1722):20160116. doi: 10.1098/rstb.2016.0116 (PMC5413868; doi:10.1098/rstb.2016.0116)
Supplement: Supplementary material: Pathogen identification and ecology [file rstb20160116supp1.docx]

**SI 1: Pathogen identification and ecology**

The identity and ecology of the specific strains of pathogens found here are not well known, and thus all analyses and discussion are kept at genus level. However, here we use our best knowledge about the natural history and identify several of these specific strains.

***Anaplasma*** – Except for one poor quality sequence, all *Anaplasma* sequences were 99-100% identical to *Anaplasma* sp. strain AnAj360 (GenBank accession AF497580.1). Unfortunately, this marker cannot accurately resolve species and the reference databases are quite confused. Among the closest named species was *Anaplasma platys* isolate 3ax1’ (GenBank KJ659045.1), which differed by one to two nucleotides. *Anaplasma platys* causes canine anaplasmosis and is present in Kenya [1]. It is generally transmitted by the brown dog tick (*Rhipicephalus sanguineus*) which is regionally present. While this species of tick generally prefers large mammal hosts, it has also been known to parasitize small mammals in other systems [2,3]. *Anaplasma platys* has previously been detected in rodents and shrews as well as in ticks collected from them [4]. However, we emphasize that the ecology of *A. platys* may or may not be relevant to the strain identified here, given the lack of resolution in the database. Other species of *Anaplasma* present within the region in Kenya include *Anaplasma ovis* [5]*,* and *Anaplasma marginale* [6].

***Bartonella -***. For *Bartonella*, we identified at least 17 major strains (some with substrains) in the 226 sequenced PCR products. *Bartonella* is an often rodent-associated pathogen whose vectors include ticks, sandflies, and fleas [7] and can infect a wide range of mammals, including humans [8]. Many *Bartonella* species have been detected in Kenya, including *B. tribocorum, B. quenslandensis, B. rochalimae*, *B. grahamii*, *B. elizabethae*, and several other unidentified genotypes of *Bartonella* [9,10].

***Borrelia –*** All *Borrelia* sequences had 100% nucleotide identity to *Borrelia burgdorferi*. However, flagellin is a conserved genetic region. There have been very few cases of Lyme disease (caused by *B. burgdorferi*) in Kenya, but two cases were reported in 2005 [11]. Other species of *Borrelia* reported regionally include *Borrelia duttonni*, *B. crocidurae*, and *B. recurrentis* (potentially just an ecotype of *B. duttonni*)[11-15], all of which use rodents as reservoir hosts. All of these latter species of *Borrelia* utilize soft ticks (primarily in the *Ornithodoros* genus) as vectors. Some of these species are known to cause relapsing fever in humans [14].

***Theileria*** – We identified only one *Theileria* strain in this study. It was most closely related to *T. equi* (89% similarity)*. Thieileria equi* causes equine piroplasmosis, which is known to affect zebras and donkeys in northern Kenya. It uses *Hyalomma* tick species as vectors [16]. Several species of *Hyalomma* ticks were found on small mammals in this study. However, 89% similarity is rather distant so it is unclear if this would be indicative of the ecology of this *Theileria* strain.

***Hepatozoon*** - Two major strains, both with substrains, were found of *Hepatozoon*. This included one strain most closely related to *H. arygbor* and *H. erhardovae*, and the other most closely related to *H. felis* and *H. ursi* (Fig SI 2). *Hepatozoon* transmission*,* unlike most vector borne pathogens, is completed by the ingestion of an ectoparasite (including ticks, fleas, mites, and lice) infected with *Hepatozoon* oocysts. Many *Hepatozoon* species have been found in Kenya; those genetically identified include *H. canis* and *H. felis* [18]

SI Table 1: Land use change effects on species richness and diversity across sites including the total number of species found across a land-use type, the average species richness per site, and the average Shannon diversity per site. Note, because sampling efforts were not equal across all land-use types, the total number of species captured should not be directly compared. Further analysis of the effects of land-use on species diversity can be found in [19]

| Land Use | Total number of species | Average species richness | Average Shannon diversity |
| --- | --- | --- | --- |
| Conserved | 25 | 5.22 | 1.10 |
| Exclosure | 18 | 7.83 | 1.38 |
| Pastoral | 17 | 4.24 | 1.17 |
| Cropland | 17 | 4.38 | 1.02 |

SI Table 2: Effects of land use, rainfall and their interaction on the five focal pathogens

|  |  |  | **Land use effects** | |  | **Main effects** | |  | **Interaction effects** | |  |
| --- | --- | --- | --- | --- | --- | --- | --- | --- | --- | --- | --- |
|  |  |  |  |  |  |  |  |  |  |  |  |
| ***ANAPLASMA*** | | AIC, df | 300.6, 45 |  |  | 302.7, 45 |  |  | 304.2, 45 |  |  |
|  |  |  | ***Z*** | ***SE*** | ***p*** | ***Z*** | ***SE*** | ***p*** | ***Z*** | ***SE*** | ***p*** |
|  | **Intercept** |  | **-2.14** | **0.2** | **<0.0001** | -1.79 | 1.12 | 0.12 | -2.07 | 1.14 | 0.08 |
|  | **Land use** | **Cropland** | **1.78** | **0.29** | **<0.0001** | 1.8 | 0.29 | 0 | 1.41 | 2.11 | 0.51 |
|  |  | Exclosure | 0.51 | 0.3 | 0.1 | 0.5 | 0.3 | 0.1 | -1.78 | 1.62 | 0.28 |
|  |  | **Pastoral** | **0.65** | **0.32** | **<0.01** | 0.64 | 0.23 | <0.01 | 5.98 | 2.07 | 0.01 |
|  | Rainfall |  |  |  |  | 0 | 0.002 | 0.75 | 0.000 | 0.002 | 0.99 |
|  | **Interactions** | Rainfall: **Cropland** | |  |  |  |  |  | 0.000 | 0.003 | 0.89 |
|  |  | Rainfall:Exclosure | |  |  |  |  |  | 0.004 | 0.003 | 0.18 |
|  |  | **Rainfall:Pastoral** | |  |  |  |  |  | **-0.090** | **0.004** | **0.01** |
|  |  |  |  |  |  |  |  |  |  |  |  |
| ***BORRELIA*** | | AIC, df | 268.9, 45 |  |  | 270.4, 45 |  |  | 268.7, 45 |  |  |
|  |  |  | ***Z*** | ***SE*** | ***p*** | ***Z*** | ***SE*** | ***p*** | ***Z*** | ***SE*** | ***p*** |
|  | **Intercept** |  | **-2.24** | **0.15** | **<0.0001** | -2.49 | 0.77 | <0.01 | -2.3 | 0.87 | 0.01 |
|  | **Land use** | **Cropland** | **1.13** | **0.28** | **<0.001** | 1.11 | 0.28 | <0.001 | 0.07 | 1.91 | 0.97 |
|  |  | **Exclosure** | **0.65** | **0.28** | **0.02** | 0.67 | 0.28 | 0.02 | 1.98 | 1.39 | 0.16 |
|  |  | **Pastoral** | **-0.48** | **0.23** | **0.04** | -0.49 | 0.23 | 0.04 | -1.89 | 1.93 | 0.33 |
|  | Rainfall |  |  |  |  | 0 | 0 | 0.74 | 0.000 | 0.000 | 0.93 |
|  | Interactions | Rainfall: Cropland | |  |  |  |  |  | 0.002 | 0.000 | 0.59 |
|  |  | Rainfall:Exclosure | |  |  |  |  |  | 0.000 | 0.000 | 0.35 |
|  |  | Rainfall:Pastoral | |  |  |  |  |  | 0.000 | 0.000 | 0.47 |
|  |  |  |  |  |  |  |  |  |  |  |  |
|  |  |  |  |  |  |  |  |  |  |  |  |
|  |  |  | **Land use effects** | |  | **All main effects** | |  | **Interaction effects** | |  |
|  |  |  |  |  |  |  |  |  |  |  |  |
| ***BARTONELLA*** | | AIC, df | 220.8, 45 |  |  | 227.2, 45 |  |  | 236.6, 45 |  |  |
|  |  |  | ***Z*** | ***SE*** | ***p*** | ***Z*** | ***SE*** | ***p*** | ***Z*** | ***SE*** | ***p*** |
|  |  |  |  |  |  |  |  |  |  |  |  |
|  | **Intercept** |  | **0.37** | **0.11** | **<0.01** | -0.34 | 0.59 | 0.57 | -0.93 | 0.72 | 0.2 |
|  | **Land use** | **Cropland** | **0.52** | **0.22** | **0.02** | 0.48 | 0.23 | 0.04 | 1.97 | 1.64 | 0.24 |
|  |  | **Exclosure** | **0.66** | **0.22** | **<0.01** | 0.66 | 0.23 | 0.01 | -1.98 | 1.25 | 0.14 |
|  |  | Pastoral | -0.15 | 0.18 | 0.41 | -0.1 | 0.19 | 0.6 | 3.63 | 1.66 | 0.04 |
|  | Rainfall |  |  |  |  | 0.001 | 0.001 | 0.24 | 0.002 | 0.001 | 0.09 |
|  | **Interactions** | Rainfall: **Cropland** | |  |  |  |  |  | 0.000 | 0.002 | 0.37 |
|  |  | Rainfall:Exclosure | |  |  |  |  |  | 0.005 | 0.002 | 0.06 |
|  |  | **Rainfall:Pastoral** | |  |  |  |  |  | **0.007** | **0.003** | **0.03** |
|  |  |  |  |  |  |  |  |  |  |  |  |
| ***THEILERIA*** | | AIC, df |  |  |  | 395.2, 45 |  |  | 284.6, 45 |  |  |
|  |  |  | ***Z*** | ***SE*** | ***p*** | ***Z*** | ***SE*** | ***p*** | ***Z*** | ***SE*** | ***p*** |
|  |  |  |  |  |  |  |  |  |  |  |  |
|  | **Intercept** |  |  |  |  | **395.2** | **2.29** | **<0.0001** | -13.12 | 0.99 | <0.001 |
|  | **Land use** | **Cropland** |  |  |  | **3.1** | **0.38** | **<0.0001** | 7.52 | 1.98 | <0.01 |
|  |  | **Exclosure** |  |  |  | **86** | **0.4** | **0.04** | 7.18 | 1.53 | <0.001 |
|  |  | Pastoral |  |  |  | 0.37 | 0.3 | 0.23 | 4.01 | 1.95 | 0.05 |
|  | **Rainfall** |  |  |  |  | **0.01** | **0** | **<0.01** | 0.016 | 0.002 | <0.0001 |
|  | **Interactions** | **Rainfall: Cropland** | |  |  |  |  |  | **-0.008** | **0.003** | **<0.01** |
|  |  | **Rainfall:Exclosure** | |  |  |  |  |  | **-0.012** | **0.003** | **<0.001** |
|  |  | Rainfall:Pastoral | |  |  |  |  |  | -0.006 | 0.004 | 0.884 |
|  |  |  |  |  |  |  |  |  |  |  |  |
|  |  |  | **Land use effects** | |  | **Main effects only** | |  | **Interaction effects** | |  |
|  |  |  |  |  |  |  |  |  |  |  |  |
| ***HEPATOZOON*** | | AIC, df | 283.6, 45 |  |  | 285.7, 45 |  |  | 288.4, 45 |  |  |
|  |  |  | ***Z*** | ***SE*** | ***p*** | ***Z*** | ***SE*** | ***p*** | ***Z*** | ***SE*** | ***p*** |
|  |  |  |  |  |  |  |  |  |  |  |  |
|  | **Intercept** |  | **-0.87** | **0.16** | **<0.0001** | -0.89 | 0.84 | 0.29 | -0.82 | 0.96 | 0.4 |
|  | **Land use** | **Cropland** | **0.61** | **0.3** | **0.05** | 0.61 | 0.31 | 0.06 | 0.34 | 2.14 | 0.88 |
|  |  | **Exclosure** | **1.13** | **1.13** | **<0.001** | 1.13 | 0.30 | <0.001 | -1.18 | 1.59 | 0.46 |
|  |  | Pastoral | -0.35 | -0.35 | 0.17 | -0.35 | 0.25 | 0.18 | 0.6 | 2.17 | 0.78 |
|  | Rainfall |  |  |  |  | 0.000 | 0.001 | 0.99 | -0.001 | 0.002 | 0.95 |
|  | Interactions | Rainfall: **Cropland** | |  |  |  |  |  | 0.000 | 0.003 | 0.90 |
|  |  | Rainfall:Exclosure | |  |  |  |  |  | 0.004 | 0.003 | 0.17 |
|  |  | Rainfall:Pastoral | |  |  |  |  |  | -0.002 | 0.004 | 0.66 |

SI Table 3: Effects of diversity and density on abundance of infected animals for each pathogen

| ANAPLASMA |  |  | *Z* | *SE* | *p* |
| --- | --- | --- | --- | --- | --- |
|  | Intercept |  | -2.35 | 0.27 | <0.0001 |
|  | Driver | Diversity | 0.39 | 0.21 | 0.07 |
|  |  | **Density** | 2.86 | 0.64 | **<0.001** |
|  |  |  |  |  |  |
| BORRELIA |  |  | *Z* | *SE* | *p* |
|  | Intercept |  | -3.1 | 0.25 | <0.0001 |
|  | Driver | Diversity | 0.31 | 0.19 | 0.11 |
|  |  | **Density** | 3.36 | 0.6 | **<0.0001** |
|  |  |  |  |  |  |
| BARTONELLA |  |  | *Z* | *SE* | *p* |
|  | Intercept |  | -0.69 | 0.17 | <0.001 |
|  | Driver | **Diversity** | 0.27 | 0.13 | **0.04** |
|  |  | **Density** | 3.85 | 0.41 | **<0.0001** |
|  |  |  |  |  |  |
| THEILERIA |  |  | *Z* | *SE* | *p* |
|  | Intercept |  | -5.9 | 0.59 | <0.0001 |
|  | Driver | Diversity | 0.34 | 0.35 | 0.34 |
|  |  | **Density** | 6.29 | 1.05 | **<0.0001** |
|  |  |  |  |  |  |
| HEPATAZOON |  |  | *Z* | *SE* | *p* |
|  | Intercept |  | -2.38 | 0.21 | <0.0001 |
|  | Driver | **Diversity** | 0.64 | 0.16 | **<0.0001** |
|  |  | **Density** | 3.84 | 0.5 | **<0.0001** |

SI Table 4: Effects of land use, rainfall and their interaction on the flea abundance

|  |  |  | Main effects only | |  | Interaction effects model | |  |
| --- | --- | --- | --- | --- | --- | --- | --- | --- |
|  |  |  |  |  |  |  |  |  |
| FLEAS | AIC, df |  | 289.18, 45 |  |  | 302.79, 45 |  |  |
|  |  |  | *Z* | *SE* | *p* | *Z* | *SE* | *p* |
|  | **Intercept** |  | 1.944 | 0.88 | **0.03** | -1.33 | 1.11 | 0.23 |
|  | Landuse | Cropland | -0.31 | 0.3 | 0.31 | 4.88 | 2.16 | 0.04 |
|  |  | Exclosure | 0.47 | 0.3 | 0.12 | -2.014 | 1.64 | 0.22 |
|  |  | Pastoral | 0.15 | 0.25 | 0.55 | 8.04 | 2.16 | <0.01 |
|  | Rainfall |  | 0.002 | 0.001 | 0.18 | 0.007 | 0.002 | <0.01 |
|  | **Interactions** | **Rainfall:Cropland** | |  |  | -0.010 | 0.003 | **0.02** |
|  |  | Rainfall:Exclosure | |  |  | 0.005 | 0.003 | 0.12 |
|  |  | **Rainfall:Pastoral** | |  |  | -0.014 | 0.004 | **<0.001** |

Fig SI 1: GARLI tree depicting the 17 major strains of *Bartonella* identified across the rodent samples.

Fig SI 2: GARLI tree depicting the phylogenetic position of the two major *Hepatozoon* lineages found in this study in comparison to other strains found on GenBank (in black). Red: strain most similar to *H.felis* and *H. ursi*; Blue: strain most similar to *H. ayorgbor* and *H. erhardovae.*

Fig SI 3: For three pathogens – *Anaplasma* (A), *Theileria* (B), and *Hepatozoon* (C) there was modest support for a positive relationship between diversity of small mammals at a site and the number of infected animals per site for any pathogen, after correction for spatial autocorrelation. The other two pathogens showed no relationship. Using site level species richness instead of Shannon diversity produces qualitatively similar (but stronger) effects.

Fig SI 4: There was no significant relationship between site level Shannon diversity and site level density of small mammals in any land use type.

Fig SI 5: Density of infected small mammals was positively correlated with the abundance (measured as number of animals per trap night) of small mammals; however this effect was stronger in cropland sites where there were a higher proportion of infected animals.

Fig SI 6: Flea abundance results closely paralleled those seen for *Bartonella,* with the number of fleas decreasing with rainfall in pastoral sites but increasing with rainfall in conserved sites.


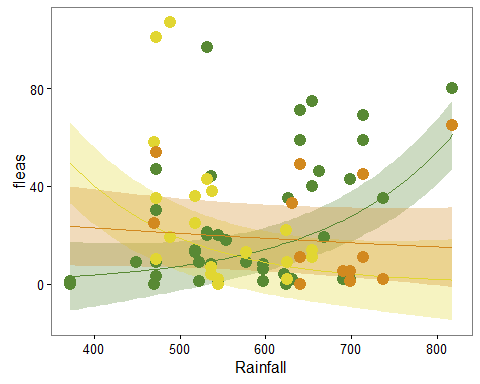


1. Matei, I. A. et al. 2016 Molecular detection of Anaplasma platys infection in free-roaming dogs and ticks from Kenya and Ivory Coast. *Parasit. Vectors* **9**, 157. (doi:10.1186/s13071-016-1443-3)

2. Winkel, K. T., Ribeiro, P. B., Antunes, L. O., Cárcamo, M. C. & Vianna, E. E. S. 2014 Rhipicephalus sanguineus sensu lato (Ixodidae) in synantropic rodents in Rio Grande do Sul, Brazil. *Rev. Bras. Parasitol. Veterinária Braz. J. Vet. Parasitol. Órgão Of. Colégio Bras. Parasitol. Veterinária* **23**, 276–279.

3. Dantas-Torres, F. 2010 Biology and ecology of the brown dog tick, Rhipicephalus sanguineus. *Parasit. Vectors* **3**, 26. (doi:10.1186/1756-3305-3-26)

4. Chae, J.-S., Yu, D.-H., Shringi, S., Klein, T. A., Kim, H.-C., Chong, S.-T., Lee, I.-Y. & Foley, J. 2008 Microbial pathogens in ticks, rodents and a shrew in northern Gyeonggi-do near the DMZ, Korea. *J. Vet. Sci.* **9**, 285–293. (doi:10.4142/jvs.2008.9.3.285)

5. Ndung’u, L. W., Aguirre, C., Rurangirwa, F. R., McElwain, T. F., McGuire, T. C., Knowles, D. P. & Palmer, G. H. 1995 Detection of *Anaplasma ovis* infection in goats by major surface protein 5 competitive inhibition enzyme-linked immunosorbent assay. *J. Clin. Microbiol.* **33**, 675–679.

6. Palmer, G. H., Barbet, A. F., Musoke, A. J., Katende, J. M., Rurangirwa, F., Shkap, V., Pipano, E., Davis, W. C. & Mcguire, T. C. 1988 Recognition of conserved surface protein epitopes on *Anaplasma centrale* and *Anaplasma marginale* isolates from Israel, Kenya and the United states. *Int. J. Parasitol.* **18**, 33–38. (doi:10.1016/0020-7519(88)90033-1)

7. Billeter, S. A., Levy, M. G., Chomel, B. B. & Breitschwerdt, E. B. 2008 Vector transmission of *Bartonella* species with emphasis on the potential for tick transmission. *Med. Vet. Entomol.* **22**, 1–15. (doi:10.1111/j.1365-2915.2008.00713.x)

8. Breitschwerdt, E. B. & Kordick, D. L. 2000 *Bartonella* infection in animals: carriership, reservoir potential, pathogenicity, and zoonotic potential for human infection. *Clin. Microbiol. Rev.* **13**, 428–438. (doi:10.1128/CMR.13.3.428-438.2000)

9. Young, H. S. et al. 2014 Declines in large wildlife increase landscape-level prevalence of rodent-borne disease in Africa. *Proc. Natl. Acad. Sci.* **111**, 7036–7041. (doi:10.1073/pnas.1404958111)

10. Halliday, J. E. B., Knobel, D. L., Agwanda, B., Bai, Y., Breiman, R. F., Cleaveland, S., Njenga, M. K. & Kosoy, M. 2015 Prevalence and diversity of small mammal-associated *Bartonella* species in rural and urban Kenya. *PLoS Negl. Trop. Dis.* **9**. (doi:10.1371/journal.pntd.0003608)

11. Jowi, J. O. & Gathua, S. N. 2005 Lyme disease: report of two cases. *East Afr. Med. J.* **82**. (doi:10.4314/eamj.v82i5.9318)

12. CDC 2015 Tick-borne Relapsing Fever.

13. Rebaudet, S. & Parola, P. 2006 Epidemiology of relapsing fever borreliosis in Europe. *FEMS Immunol. Med. Microbiol.* **48**, 11–15. (doi:10.1111/j.1574-695X.2006.00104.x)

14. Lucas, A. O. & Gilles, H. M. 2003 *Short Textbook of Public Health Medicine for the Tropics*. CRC Press.

15. Cutler, S. J. Cutler, Bonilla, E. M., Singh, R. J. 2010. Population structure of East African relapsing fever *Borrelia* spp. *Emerging Infectious Diseases* **16,** 1076-1080.

16. Hawkins, E. et al. 2015 Prevalence of theileria equi and babesia caballi as well as the identification of associated ticks in sympatric grevy’s zebras (equus grevyi) and donkeys (equus africanus asinus) in northern kenya. *J. Wildl. Dis.* **51**, 137–147. (doi:10.7589/2013-11-316)
